# Number of Births and Risk of Diabetes in China's Older Women

**DOI:** 10.3389/fmed.2022.798787

**Published:** 2022-04-26

**Authors:** Ying-wen Gu, Shuo Zhang, Jia-hao Wang, Hua-lei Yang, Si-qing Zhang, Yi-dan Yao, Yuan-yang Wu, Lin Xie, Zhi-yun Li, Jin-yan Cao

**Affiliations:** ^1^School of Public Administration, Zhongnan University of Economics and Law, Wuhan, China; ^2^Institution of Population and Labor Economics, University of Chinese Academy of Social Science, Beijing, China; ^3^College of Politics and Public Administration, Qingdao University, Qingdao, China

**Keywords:** number of birth, risk of diabetes, reproductive behavior, older women, health

## Abstract

As an important life event in individuals' life, childbirth will affect the health of women to different degrees. More and more attention has been paid to whether the number of births will affect the incident diabetes in elderly women, but there are few related studies. Based on the data of the Chinese Longitudinal Healthy Longevity Survey in 2018, 6,159 older women are selected as the study population. Logistic regression analyses are used to estimate the relationship between the number of births and diabetes risk. For each additional birth, the odds ratio of maternal diabetes will decrease by 6.9% and the result is significant at the 1% level, especially among mothers having four children or less. The conclusion is equally applicable in the sample of fathers and urban mothers, but the increase in the number of births will increase the risk of diabetes in rural mothers, although this result is not statistically significant. Later age at first birth, later age at last birth, the longer childbearing period, and birth interval will significantly reduce the risk of diabetes.

## Introduction

Under the strategy of “healthy China,” we need to adhere to prevention first and promote a healthy and civilized lifestyle to reduce the incidence of diseases. We should focus on solving the health problems of women, children, the elderly, and other key groups. Childbirth is a unique life experience for women and its impact on women's physical health has been a hot issue. During pregnancy, women are prone to change their diets, increase their energy intake, and reduce the intensity of physical activity. These changes may affect women's health, including insulin resistance, fat accumulation, redistribution, dyslipidemia, and inflammation, especially the risk of developing diabetes and other cardiometabolic diseases in their future lives (1). Diabetes, as one of the common chronic diseases, is a metabolic disease characterized by hyperglycemia and can be said to be a lifelong disease. When individuals are diagnosed with diabetes, it can have an impact on their own physical and psychological health, manifesting itself in symptoms such as overeating, wasting, obesity, fatigue, irritability, and anger. Theoretically, childbirth significantly affects physiological indicators such as metabolism, insulin secretion, and insulin sensitivity in women, which in turn is associated with diabetes. Therefore, it is of great theoretical and practical importance to explore the relationship between the number of births and the risk of diabetes in mothers.

Many scholars have conducted numerous studies on the relationship between reproductive behaviors and diabetes risk. There are some conflicting findings regarding the relationship. Middleton and Caird (2) pointed out that mothers' risk of diabetes increases with the number of births. Specifically, having one child increased a woman's risk of diabetes by 20%, three children by 100%, and six or more children by 400%. Subsequently, researchers in different countries conducted similar studies with women in their own countries to test the applicability of the finding in their own countries. In the study of Hispanic women, Chinese Americans, Southeast Asian, and Danish women in Columbia, researchers have come to a similar conclusion that increasing the number of births would increase the risk of maternal diabetes (3–7). Scholars have shown that although the increase in the number of births increased the diabetes risk, further studies showed that there was a negative correlation between the number of births and maternal mortality (8). When other factors were taken into consideration, there was uncertainty about whether there was a positive correlation between the number of births and the risk of diabetes. For example, in a large sample study of American women, the relationship was diminished and became insignificant when scholars adjusted the variable of weight gain (9). Other researchers have adjusted the age and waist circumference of the sample and found that the number of births significantly reduced the risk of diabetes in women (10).

In the childbearing period, a shorter childbearing period was associated with a higher risk of diabetes (11). The existing studies on the specific relationship between reproduction and the risk of diabetes were inconsistent. Some studies have shown that there is a U-type relationship between the childbearing period and the risk of type 2 diabetes (12, 13). Too short or too long, a childbearing period can increase a woman's risk of developing diabetes. Studies have also shown that the long childbearing period was a simple positive correlation with the risk of diabetes (9, 14).

Studies have shown that the relationship between other specific reproductive behaviors such as abortion and early pregnancy and diabetes was complex. Miscarriage or recurrent miscarriage was a significant risk factor for type 2 diabetes, both of which increased the likelihood that a woman developed diabetes later in life (15). Miscarriage also influenced the positive association between the number of births and the risk of diabetes. In one cohort study, the positive association between the number of children (>4) and the risk of diabetes disappeared when abortion was taken into account (16).

In addition to explore the relationship between the number of births and other reproductive behaviors and diabetes risk, scholars from various countries were also exploring the possible reasons behind these relationships. In biological mechanisms, insulin resistance, insulin sensitivity, and metabolic syndrome were all the important triggers of diabetes. Reduced insulin sensitivity led to insulin resistance, which increased the likelihood of developing diabetes, while metabolic syndrome was also an important trigger of diabetes. Pregnancy could lead to a state of insulin resistance in a woman's peripheral tissues, which may be severe enough to lead to gestational diabetes in susceptible non-diabetic women. It was generally accepted that pregnancy-related insulin resistance resolved after delivery, but small metabolic changes may persist, leading to an increased risk of future diabetes (17). In terms of the number of births, an increase in the number of births may have some effects on the development of metabolic syndrome later in life (18). For the childbearing period, one study found that the short childbearing period increased insulin resistance levels (19). In addition, abortion significantly increased the likelihood of insulin dependence (20).

Based on the above findings, it is worth further research and discussion as to whether they still hold true for the Chinese female sample. Therefore, the study uses data from the Chinese Longitudinal Healthy Longevity Survey (CLHLS) in 2018 to conduct a regression analysis using a logistic regression model with older women aged 65 years and above as the study population. Compared to previous studies, the aim of this study was to focus on the effect of the number of births on diabetes risk in later life in Chinese women. This study seeks to find the sex of the child as an instrumental variable to obtain more robust and reliable findings. In addition to the number of births, this study also examines the association between other reproduction behaviors and maternal risk of diabetes, further enriching the research experience with Chinese samples and diving the reasonable analysis of social mechanisms.

## Methods

### Data

The data of this study come from the Chinese Longitudinal Healthy Longevity Survey in 2018. The survey covers 23 provinces and autonomous regions in China, with aged 65 and above and 35–64-year-old adult children as the survey object, and is the earliest and longest social science survey in China. The contents of questionnaire for the surviving interviewees include the basic information of the elderly and their families, self-evaluation of health and quality of life, personality and psychological characteristics, disease treatment, and lifestyle, etc. Combined with the research content of this paper, the elderly women aged 65 and above are selected as the research objects. After excluding the variables and missing values unrelated to this study, the final sample number is 6,159 including 2,680 rural women samples, accounting for 43.51% of the total sample, and 3,479 urban female samples, accounting for 56.49%.

### Variable Definitions

#### Dependent Variable

The risk of diabetes in older women is selected as the dependent variable. The number of births and other reproductive behaviors may affect incident diabetes in later life by some physiological channel or social channel. In the questionnaire, respondents are asked to self-report whether or not they have diabetes, and this is combined with whether or not they have been diagnosed in a hospital to obtain the variable of whether or not they have diabetes. It is set as a dummy variable, in which having diabetes is assigned a value of 1 and others are assigned a value of 0.

#### Independent Variable

The number of births, which measures the number of individual deliveries, is selected as the core explanatory variable of this paper. Here, it should be noted that the number of adopted children is not included. In the questionnaire, the respondents will be asked “How many children have you had in your life (including those who died)?”. The data of this problem constitute the independent variable of this paper. In the selected sample, the vast majority of respondents have six or less children, which is consistent with the reality. To further explore the influence of other reproductive behaviors, age at first birth, age at last birth, childbearing period, and birth interval are added as the supplements.

#### Control Variable

Based on the research content, centering on the health status of older people and referring to some scholars (21), this paper includes the participants' age, residence, education, spouse, living standard, income, smoking status, alcohol drinking, physical activity, and sugar intake into the control variables. We set residence, education, and spouse as the dummy variables. For residence, the participants living in cities and towns are assigned to 1 and those living in rural areas are assigned to 0. Illiterate is assigned a value of 1, other is assigned a value of 0. Those who live with their spouse are assigned to 1. For other control variables, answers to sugars and living standard are assigned, respectively, to integers between 1 and 5. The smaller the number, the more frequent sugars intake and the better the living standard is. Respondents' responses on smoking, drinking, and physical activity in their past and present lives were collated separately to obtain the corresponding variables. Never drinking is assigned a value of 1, past drinking but not currently drinking is assigned a value of 2, past not drinking but currently drinking is assigned a value of 3, and past and current drinking is assigned a value of 4. Similar settings for smoking status and physical activity are described above.

**Figure 1:**
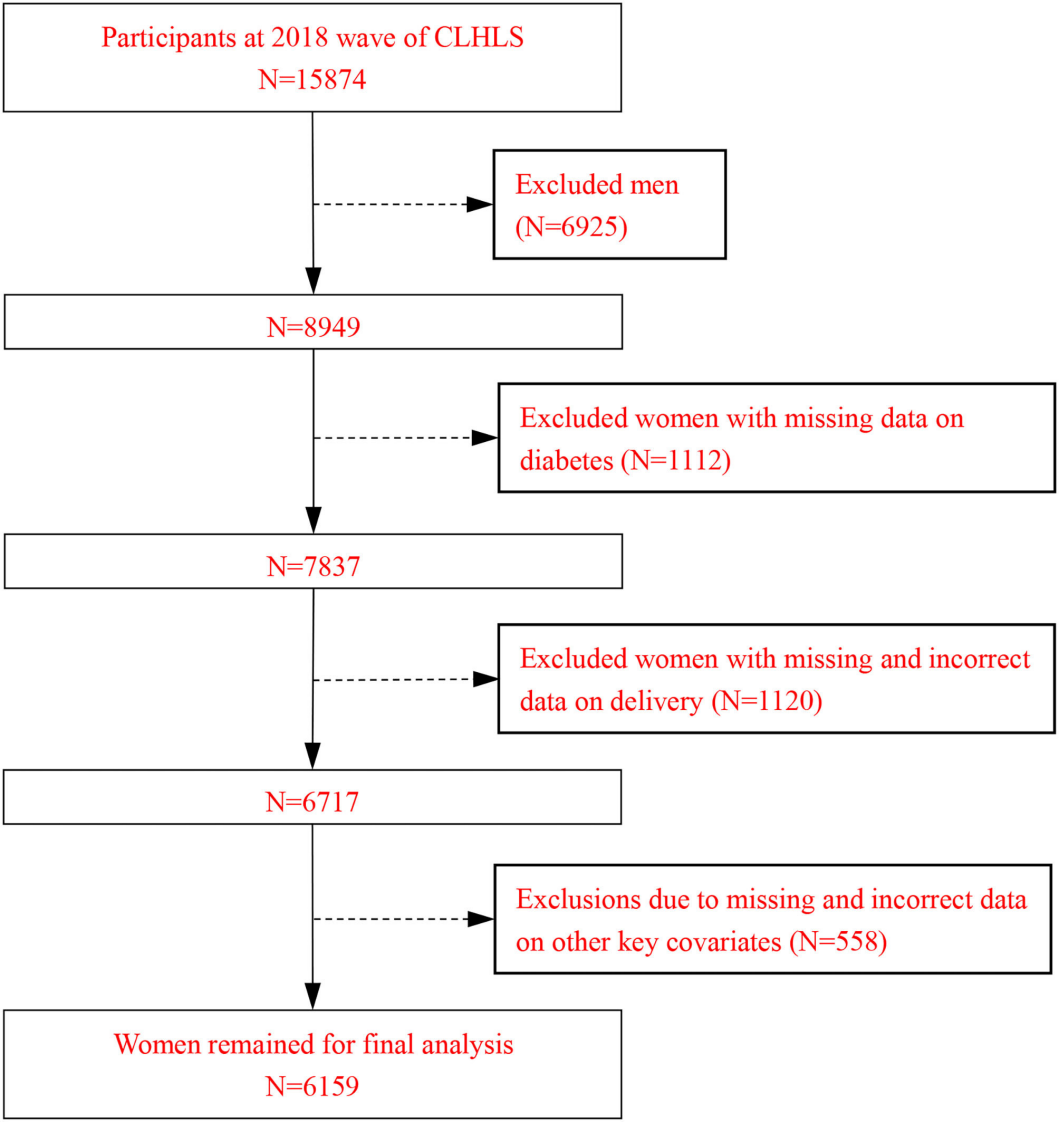
Flow chart of the study population selection process.

Figure 1 shows specifically the selection process for the study population. To obtain relatively robust analysis results, this study treats the missing values as follows: if the sample has <3% of missing data for a variable, we choose to remove the sample; if the percentage is more than 3%, we interpolate using interpolation and extrapolation methods.

### Model Setting

Because the dependent variable is set to a binary variable, a logistic regression model is selected for the analysis. The model is as follows:


(1) $$\begin{array}{r} Diabetes_{it}=\alpha_{0}+\alpha_{1}CB_{it}+\alpha_{2}X_{it}+\phi_{it} \end{array}$$

In equation (1), *Diabetes*_*it*_ indicates the risk of diabetes of the participants in a certain period of time; *CB*_*it*_ (childbearing behavior) refers to the reproductive behavior in a certain period of time, including the number of births, age at first birth, age at last birth, childbearing period, and birth interval; *X*_*it*_ represents other control variables; ϕ_*it*_ represents the random error term; α_1_ is the coefficient to be estimated in this paper, which reflects the impact of the number of births and other reproductive behaviors on diabetes in elderly women.

## Results

### Descriptive Analyses

Descriptive results of related variables are shown in Table 1. For the dependent variable, 10.50% of the whole sample suffer from diabetes. In terms of urban and rural distribution, the risk of diabetes in older women in urban areas is higher than that in rural older women. As for the independent variables, the average number of births in the sample is about 4, and the number of births of rural women is higher than that of urban women. In addition to consider the number of births, we also pay attention to the impact of other reproductive behaviors such as age at first birth, age at last birth, birth interval, and childbearing period on diabetes. The average age at the first child in the whole sample is about 23.01; the average age at the last child is about 34.88; the average childbearing period is about 11.90 years, and the reproductive period of urban women will be relatively shorter; the average birth interval is 2.68 years.

**Table 1:** Descriptive statistics.

**Variables**	**Mean of all samples/%**	**Rural samples**		**Urban samples**		**Mean-Diff**	**T_value**
		***N***	**Mean**	***N***	**Mean**		
**Dependent variable**							
Diabetes (1 = yes)	10.50%	2,680	0.064	3,479	0.137	−0.073^***^	−9.329
**Independent variables**							
Number of births	4.16	2,680	4.384	3,479	3.982	0.402^***^	7.583
Age at first birth	23.01	2,490	22.739	3,253	23.215	−0.476^***^	−4.217
Age at last birth	34.88	2,423	35.524	3,152	34.378	1.146v^***^	6.000
Childbearing period	11.90	2,399	12.806	3,123	11.199	1.607^***^	8.010
Birth interval	2.68	2,398	2.795	3,123	2.591	0.204^***^	4.809
**Control variables**							
Age	87.21	2,680	87.597	3,479	86.913	0.684^**^	2.182
Residence (1 = urban)	56.49%	2,680	0.738	3,479	0.587	0.150^***^	12.446
Education (1 = illiteracy)	65.27%	2,680	0.274	3,479	0.274	−0.000	−0.029
Spouse (1 = yes)	27.41%	2,680	2.985	3,479	2.890	0.094^***^	5.977
Living standard	2.93	2,680	9.384	3,479	10.078	−0.694^***^	−14.952
Income	9.78	2,680	1.163	3,479	1.154	0.009	0.609
Smoking status	1.16	2,680	1.228	3,479	1.229	−0.001	−0.040
Alcohol drinking	1.23	2,680	1.566	3,479	2.011	−0.445^***^	−14.279
Physical activity	1.82	2,680	3.591	3,479	3.564	0.028	0.739
Sugar intake	3.58	2,680	87.597	3,479	86.913	0.684^**^	2.182

^*^ *p < 0.10,*

** *p < 0.05,*

*** *p < 0.01*.

In terms of control variables, the average age is 87.21 years, more than half of the women (56.49%) live in cities and towns, 65.27% of them are illiteracy, 32.65% of them live with their spouses, the vast majority (91.83%) have been non-smokers, and 88.78% have been non-drinkers.

### Regression Results

Table 2 reports logistic regression results of the impact of the number of births on maternal diabetes. Model 1 shows the regression results when no control variables are included, and the odds ratio for diabetes is 0.843 (*p* < 0.01). Taking into account the effect of confounding factors, models 2 to 4 show the regression results when other variables are added in turn, respectively. As shown in model 2, controlling for age, residence, and spousal status of the sample, the odds ratio for diabetes is 0.915 (*p* < 0.01). When the lifestyle habits of the sample are further taken into account, the result of model 3 shows that for each additional child, the odds ratio of diabetes decreases by 9.2% (*p* < 0.01). Finally, model 4 takes into account the socioeconomic status of the sample, and the results show that for each additional child, the odds ratio of diabetes decreases by 6.9% (*p* < 0.01). The risk of diabetes in older women aged 90 and above will increase significantly compared to the 65–69-year-old women. Older women living in urban or higher-income level have the higher risk of diabetes.

**Table 2:** Estimations of the effects of the number of births on diabetes in older women.

	**Model 1**		**Model 2**		**Model 3**		**Model 4**	
	**Coefficient**	**Odds ratio**	**Coefficient**	**Odds ratio**	**Coefficient**	**Odds ratio**	**Coefficient**	**Odds ratio**
Number of births	−0.171^***^	0.843^***^	−0.089^***^	0.915^***^	−0.096^***^	0.908^***^	−0.071^***^	0.931^***^
	(0.022)	(0.018)	(0.026)	(0.023)	(0.027)	(0.024)	(0.028)	(0.025)
Residence			0.795^***^	2.215^***^	0.784^***^	2.190^***^	0.709^***^	2.032^***^
			(0.094)	(0.212)	(0.095)	(0.214)	(0.096)	(0.203)
Spouse			0.130	1.139	0.104	1.110	0.090	1.094
			(0.101)	(0.116)	(0.102)	(0.114)	(0.103)	(0.113)
Smoking status					−0.056	0.945	−0.052	0.949
					(0.084)	(0.081)	(0.084)	(0.082)
Alcohol drinking					−0.169^**^	0.844^**^	−0.168^**^	0.845^**^
					(0.074)	(0.064)	(0.075)	(0.064)
Physical activity					0.059^*^	1.061^*^	0.031	1.031
					(0.034)	(0.036)	(0.035)	(0.036)
Sugar intake					0.343^***^	1.409^***^	0.354^***^	1.425^***^
					(0.037)	(0.051)	(0.037)	(0.052)
Education							−0.348^***^	0.706^***^
							(0.103)	(0.070)
Living standard							−0.027	0.973
							(0.078)	(0.073)
Income							0.061^**^	1.062^**^
							(0.031)	(0.030)
**Age (ref** **=** **Aged 65–69)**								
Aged 70–79			0.125	1.133	0.159	1.173	0.176	1.192
			(0.137)	(0.154)	(0.139)	(0.161)	(0.139)	(0.165)
Aged 80–89			0.005	1.005	0.094	1.099	0.181	1.198
			(0.153)	(0.152)	(0.153)	(0.168)	(0.155)	(0.186)
Aged 90–99			−0.644^***^	0.525^***^	−0.513^***^	0.599^***^	−0.404^**^	0.668^**^
			(0.175)	(0.091)	(0.177)	(0.106)	(0.183)	(0.120)
Aged 100 or above			−2.144^***^	0.117^***^	−1.968^***^	0.140^***^	−1.830^***^	0.160^***^
			(0.237)	(0.028)	(0.239)	(0.033)	(0.246)	(0.039)
_cons	−1.479^***^	0.228^***^	−1.978^***^	0.138^***^	−3.199^***^	0.041^***^	−3.636^***^	0.026^***^
	(0.090)	(0.020)	(0.163)	(0.023)	(0.267)	(0.011)	(0.509)	(0.013)
*N*	6,159	6,159	6,159	6,159	6,159	6,159	6,159	6,159
*R^2^*	0.016	0.016	0.096	0.096	0.124	0.124	0.129	0.129

* *p < 0.10,*

** *p < 0.05,*

*** *p < 0.01; standard errors are reported in parentheses*.

Further, the whole sample is divided into two sub-samples according to the average number of births. The results are shown in Table 3. In the group with less than five children, it can be found that for each additional child, the odds ratio of diabetes will be reduced by 9.9% (*p* < 0.05). In the group of mothers who have more than four children, the odds ratio of diabetes will be increased by 2.5% for every increase in the number of births, but this result is not statistically significant. Based on this, the positive effect that the number of births will reduce the risk of maternal diabetes to a certain extent is mainly reflected in the mothers having less than five children.

**Table 3:** Subgroup analysis.

	**Full sample**		**Having four children or less**		**Having five children or more**	
	**Coefficient**	**Odds ratio**	**Coefficient**	**Odds ratio**	**Coefficient**	**Odds ratio**
Number of births	−0.071^***^	0.931^***^				
	(0.028)	(0.025)				
Number of births 1			−0.105^**^	0.901^**^		
			(0.051)	(0.046)		
Number of births 2					0.024	1.025
					(0.071)	(0.069)
Control variables	Yes	Yes	Yes	Yes	Yes	Yes
*N*	6,159	6,159	3,657	3,657	2,502	2,502
*R^2^*	0.129	0.129	0.122	0.122	0.125	0.125

^*^p < 0.10,

** *p < 0.05,*

*** *p < 0.01; standard errors are reported in parentheses*.

### Sensitivity Analyses

To ensure the robustness and reliability of the results and to further verify the control of confounding factors, this paper will cut through the two perspectives of sample restrictions and variable definitions to conduct sensitivity analysis, and the results are shown in Table 4. First, the samples living in the eastern and western regions are selected separately to test the reliability of the above conclusion. The results are shown in models 5 and 6, respectively. The above conclusion is found to still hold, taking into account regional differences. The results show that having more children reduces the risk of diabetes in mothers. Second, the study will again run the regression using the sample's self-reported presence or absence of diabetes as the explanatory variable. The results of model 7 show that controlling for other variables, the number of births still reduces the odds of diabetes, with an odds ratio of 0.952 (*p* < 0.1).

**Table 4:** Sensitivity analysis results.

	**Model 5**	**Model 6**	**Model 7**
Number of births	0.932^**^	0.854^*^	0.952^*^
	(0.031)	(0.070)	(0.025)
Control variables	Yes	Yes	Yes
*N*	3,796	745	6,159
*R^2^*	0.135	0.171	0.124

*The odds ratios are shown in the above and standard errors are reported in parentheses;*

* *p < 0.10,*

** p < 0.05, ^***^p < 0.01.

### Analysis of Instrumental Variables

For possible endogeneity issues, this study has made additional efforts to control for variables, but endogeneity issues may still exist. To avoid confounding the relationship between the number of births and diabetes risk by other observed and unobserved factors, this study will look for the instrumental variables for the number of births to reduce possible endogeneity issues that may lead to biased and non-consistent logistic estimates. Table 5 reports the results of the two-stage least squares (2SLS) regression that selected the sex of a first child and having sons as the instrumental variables. The results of the first-stage regression show that the effects of both sex of a first child and having sons are significant, controlling for other variables, and that the F-values are greater than the critical value of 10, indicating that the correlation conditions for the instrumental variables are satisfied. The results of the second-stage regression showed that the coefficient on the number of births is significantly negative, controlling for other factors, in line with the logistic regression results, indicating that the finding that the number of births reduces the risk of maternal diabetes is robust.

**Table 5:** Two-stage least squares regression results.

**Variable**		**First stage**	**Second stage**
		**Number of births**	**Diabetes**
Independent variable	Number of births		−0.012^*^
			(0.007)
Instrumental variable	Sex of first child	−0.492^***^	
		(0.047)	
	Having sons	2.401^***^	
		(0.083)	
	Control variables	Yes	Yes
	*N*	6,074	6,074
	*F*	419.47^***^	35.41^***^
	R^2^	0.265	0.073

* p < 0.10, ^**^p < 0.05,

*** *p < 0.01; standard errors are reported in parentheses*.

This study will test the legitimacy of instrumental variables from two perspectives, and the results are shown in Table 6. According to Angrist and Pischke (22), legitimate instrumental variables need to satisfy both correlation and exogeneity conditions. Underidentification test is used to determine whether the instrumental variables are related to the endogenous variables. The Lagrange multiplier (LM) results show that the original hypothesis is rejected, indicating that there is a relationship between the two. Weak identification test is used to determine the strength of the relationship between the instrumental variables and the endogenous variables. The results of Cragg-Donald Wald rank test (23) show that the F-value is >10; that is, the instrumental variables have a strong correlation with the endogenous variables. Weak-instrument-robust inference test is used to determine the degree of significance of the instrumental variables themselves. The Sanderson–Windmeijer multivariate results show that the F-value is significantly >10, indicating that the instrumental variables have strong explanatory power. The exogeneity of the instrumental variables is judged by the overidentification test; that is, the instrumental variables are not correlated with the perturbation terms. The test result is not significant, indicating that the instrumental variables are exogenous.

**Table 6:** Results of reasonableness tests for instrumental variables.

**Test name**	**Result**	**Conclusion**
**First-stage**		
**Underidentification test**		
Kleibergen-Paap rk LM statistic	374.10^***^ (0.0000)	Significant (pass)
(chi-square)		
**Weak identification test**		
Cragg-Donald Wald F statistic	375.19	
Sanderson–Windmeijer multivariate	419.468^***^ (0.0000)	Significant (pass)
F test		
**Second-stage**		
**Underidentification test**		
Kleibergen-Paap rk LM statistic	374.103^***^ (0.0000)	Significant (pass)
(chi-square)		
**Overidentification test**		
Hansen J statistic (chi-square)	1.180 (0.2774)	Insignificant (pass)

^*^p < 0.10, ^**^p < 0.05,

*** *p < 0.01; standard errors are reported in parentheses*.

### Heterogeneity Analyses

In view of the possible differences in the effects of different groups and China's social characteristics of the two-element structure of urban and rural areas, the urban and rural differences and gender differences in the number of births affecting maternal diabetes will be further examined, as shown in Tables 7, 8.

**Table 7:** Estimates of urban–rural differences in the effects of the number of births on diabetes.

	**Urban samples**			**Rural samples**		
	**Model 8**	**Model 9**	**Model 10**	**Model 11**	**Model 12**	**Model 13**
Number of births	0.865^***^			1.120^**^		
	(0.029)			(0.053)		
Number of births 1 (≤ 4)		0.830^***^			1.222^*^	
		(0.048)			(0.143)	
Number of births 2 (>4)			0.951			1.156
			(0.089)			(0.118)
Control variables	Yes	Yes	Yes	Yes	Yes	Yes
N	3,479	2,161	1,318	2,680	1,496	1,184
R^2^	0.115	0.096	0.109	0.141	0.136	0.176

* *p < 0.10,*

** *p < 0.05,*

*** *p < 0.01; standard errors are reported in parentheses; the above table reports the odds ratio*.

**Table 8:** Estimates of gender differences in the effects of the number of births on diabetes.

	**Female samples**			**Male samples**		
	**Model 14**	**Model 15**	**Model 16**	**Model 17**	**Model 18**	**Model 19**
Number of births	0.931^***^			0.843^***^		
	(0.025)			(0.027)		
Number of births (≤ 4)		0.901^**^			0.869^***^	
		(0.046)			(0.045)	
Number of births (>4)			1.025			0.838
			(0.069)			(0.106)
Control variables	Yes	Yes	Yes	Yes	Yes	Yes
N	6,159	3,657	2,502	4,657	3,206	1,469
R^2^	0.129	0.122	0.125	0.091	0.076	0.073

^*^p < 0.10,

** *p < 0.05,*

*** *p < 0.01; standard errors are reported in parentheses; the above table reports the odds ratio*.

According to the results of model 8, it can be found that in urban samples, for each additional child, the odds ratio of diabetes decreases by 13.5% (*p* < 0.01). The conclusion is consistent with the conclusion of the whole sample model. However, model 11 shows that the odds ratio of diabetes increases by 12% for rural older women, for each additional child. Specifically, it can be found that the effect of the number of births on the risk of diabetes is more pronounced in groups with less than five children. Taking into account other control variables, for each additional child born, the odds ratio for diabetes in urban mothers is 0.83 (*p* < 0.01), compared to 1.222 for rural mothers. Table 8 reports the difference in diabetes risk among older women and men, respectively. Models 14 and 17 show that the increase in the number of births will reduce the risk of diabetes for fathers and mothers to varying degrees. That is to say, for each additional child, the odds ratio of diabetes for fathers will be reduced by 15.7% (*p* < 0.01). In families with less than five children, the positive effect of the number of births in reducing the risk of diabetes is greater. Specifically, for each additional child, the odds ratio for maternal diabetes is 0.901 (*p* < 0.05) and for paternal diabetes is 0.869 (*p* < 0.01). However, in families with five or more children, the positive effect is reflected in the father group.

### Further Analyses

Childbirth as an important life event has a significant impact on women's health status, and existing studies suggest that in addition to the number of births, other reproductive behaviors may also influence their risk of developing diabetes.

Table 9 reports the estimates of the effects of other reproductive behaviors on maternal diabetes risk. Model 20 shows that the older the mother's age at first birth, the higher the risk of diabetes is, but the result is not statistically significant. As shown in model 21, for every 1-year addition the age that the mother gives birth to the last child, the odds ratio of diabetes is 0.968 (*p* < 0.01). Model 22 shows that when mothers' childbearing period is extended by 1 year, their odds ratio of diabetes decreases by 2.6% (*p* < 0.01). Model 23 shows that the longer the interval between births, the lower the risk of diabetes in the mother.

**Table 9:** Estimates of the effect of other reproductive behaviors on diabetes in elderly women.

	**Model 20**	**Model 21**	**Model 22**	**Model 23**
Age at first birth	0.997			
	(0.012)			
Age at last birth		0.968^***^		
		(0.008)		
Childbearing period			0.974^***^	
			(0.008)	
Birth interval				0.941^*^
				(0.033)
Control variables	Yes	Yes	Yes	Yes
N	5,743	5,575	5,522	5,521
R^2^	0.126	0.130	0.129	0.127

* p < 0.10, ^**^ p < 0.05,

*** *p < 0.01; standard errors are reported in parentheses; the above table reports the odds ratio*.

Similarly, Table 10 reports the urban–rural differences in the impact of other reproductive behaviors on diabetes risk in older women. The results of models 24 and 28 show urban–rural differences in the effect of age at first birth on the risk of maternal diabetes, but the results are not statistically significant. Models 25 and 29 show that whether in the urban sample or in the rural sample, the impact of age at last birth on the mother's diabetes risk is the same. That is, when the mother's age at last child is delayed by 1 year, the odds ratio of diabetes for urban mothers decreases by 4.4% (*p* < 0.01), and odds ratio of diabetes for rural mothers decreases by 0.4%. The results of models 26 and 27 show that the longer the period and interval between having children, the lower the odds of diabetes in urban mothers, with odds ratios of 0.959 (*p* < 0.01) and 0.922, respectively. However, the odds ratio of diabetes in rural mothers increased in models 30 and 31.

**Table 10:** Estimates of urban–rural differences in the effects of other reproductive behaviors.

	**Urban samples**				**Rural samples**			
	**Model 24**	**Model 25**	**Model 26**	**Model 27**	**Model 28**	**Model 29**	**Model 30**	**Model 31**
Age at first birth	1.002				0.965			
	(0.013)				(0.024)			
Age at last birth		0.956^***^				0.996		
		(0.010)				(0.015)		
Childbearing period			0.959^***^				1.012	
			(0.010)				(0.015)	
Birth interval				0.922^**^				1.010
				(0.038)				(0.068)
Control variables	Yes	Yes	Yes	Yes	Yes	Yes	Yes	Yes
*N*	3,253	3,152	3,123	3,123	2,490	2,423	2,399	2,398
R^2^	0.110	0.117	0.117	0.111	0.135	0.136	0.138	0.138

*p < 0.10,

** *p < 0.05,*

*** *p < 0.01; standard errors are reported in parentheses; the above table reports the odds ratio*.

## Discussion

An increasing number of births will significantly reduce the risk of diabetes. In addition to genetic factors, the development of diabetes is often associated with environmental factors such as a high-calorie diet, poorly structured diets such as overeating, stressful exertion, and obesity due to reduced physical activity, etc. For evolutionary biology, the one-time somatic theory and maternal failure theory believe that fertility will compete with the resources required for body maintenance (24, 25). The relevant evidence shows that mothers will choose to sacrifice their nutritional intake and health investment when they are young due to the factors such as family resource constraints and parenting pressures. Furthermore, the more mother gives birth to children, the greater the pressure on parenting. Taking care of children is undoubtedly a physically exhausting activity, which will make up for their reduced exercise time due to childcare. The results of sub-sample of gender show that an increase in the number of births will significantly reduce the risk of father's diabetes. More children will undoubtedly increase the pressure of fathers. Due to limited family resources, they will face a poor diet and other living environments and thus reduce the possibility of diabetes.

An increase in the number of births will significantly reduce the risk of diabetes for urban mothers. Based on the difference in living environments, mothers living in urban often face greater pressure of life. When the number of births is larger, it means that they need to spend more energy and physical energy to take care of their children and families. At the same time, the workload will be more intense and they will need to work harder than before to earn money to support their children, which will avoid the probability of obesity caused by factors such as poor diet structure and reduced physical activity, which will in turn reduce their risk of developing diabetes. For mothers living in rural, given the unique rural lifestyle, where each family has its own land and other relatively fixed assets, the living environment is relatively easy and mothers are not under much pressure to work after giving birth. Rural mothers face far less pressure to raise their children than their urban counterparts, and as a result, obesity and hypertension become more prominent in their later years; the more children they have, the more likely they are to develop diabetes.

Physiologically, women having children earlier in life may have adverse effects on their bodies. Some scholars (26, 27) have suggested that there is a significant association between hypertension, diabetes, and chronic lung disease, which to some extent affects women's health in later life, while in contrast, having children later in life reduces the incidence of hypertension. For women who have children later in life, their endogenous estrogen production is prolonged, their age of menopause is delayed, and later pregnancy, childbirth, and breastfeeding may stimulate the female biological system, which in turn has a positive impact on their survival (21). Second, on a societal level, mothers who have children early may not be prepared with adequate resources to cope with the stress of parenting (28), whereas women who have children later in life will be more physically and psychologically matured and already have sufficient resources to easily cope with the stress of parenting. Studies have shown that the risk of diabetes is associated with individual stressors in life, such as stress, strain, and pressure. Furthermore, one study found that delaying marriage and childbearing allowed women to build up more human capital, which in turn helped to improve their health (29). Finally, the greater the number of children born to mothers means that their childbearing period is extended. This is accompanied by a decrease in physical capacity and a rise in the cost of childcare due to age and the act of giving birth, which prevents them from overfeeding. Moreover, caring for children and the family is a very exhausting and physically demanding task, which reduces the risk of diabetes to a certain extent.

Compared to previous studies, this study is innovative in that it focuses on the effect of the number of births on diabetes risk in later life in Chinese women and seeks to find the sex of the child as an instrumental variable to obtain the more robust and reliable findings. In addition to the number of births, this study also examines the association between other reproduction behaviors and maternal risk of diabetes, further enriching the research experience with Chinese samples.

We acknowledge several limitations to our study. First, the study does not take into account additional sample information, such as underlying disease conditions in old age, family genetic history, and differences in life circumstances, all of which emphasize the need for future studies to provide a more scientifically accurate understanding of the relationship between reproductive behavior and late-life diabetes in women. Second, this study fails to fully control for the confounding factors. Despite controlling for observed and unobserved confounders through regression adjustment, sensitivity analysis, and instrumental variable regression, the confounding role of confounders in this effect will be further addressed to obtain the extent to which the number of births affects the odds of developing diabetes. Then, this study is a cross-sectional study that ignores the temporal trends in the development of the effect of the number of births on diabetes risk, which also suggests a focus for future research. Finally, this study fails to adequately account for other causes that influence an individual's odds of developing diabetes in later life, which is important to obtain reliable findings.

## Conclusions

This paper selects older women aged 65 and above as the study population using the data from the Chinese Longitudinal Healthy Longevity Survey in 2018 and uses a logistic regression model to empirically examine the effects of the number of births on the risk of maternal diabetes. The results of the full-sample regression analysis show that for every increase in the number of births, the odds ratio for diabetes decreases by 6.9%, which was significant at the 1% level, which is particularly pronounced in mothers having four children or less. Heterogeneity analysis results reveal the differences in the effect of the number of births on diabetes risk. Specifically, for each additional child born, the odds ratio for diabetes decreases by 13.5% for mothers living in urban areas and is significant at the 1% level, while the odds ratio increases for rural mothers. An increase in the number of births also significantly reduces the diabetes risk in fathers. Later age at first birth, later age at last birth, and longer childbearing periods, and intervals between births all reduce the odds ratio of diabetes in older women, and that this finding is more prevalent in urban samples.

Based on the above findings, it is known that there is an association between the number of births and the risk of diabetes in older women. According to the seventh census, the population aged 60 years and above accounts for ~18.7% of the total population, with 13.5% of the population aged 65 years and above, and the population is already aging further. At the same time, the physical health of older people is attracting more and more attention, especially the health of older women. As an important life event in a woman's life, childbirth is a unique life experience for women, and the impact of this act on women's physiological health has been a hot topic of discussion and research. In addition to genetic factors, the development of diabetes is also linked to environmental factors in the life of individuals, such as obesity due to an irrational dietary structure, lack of exercise, stressful exertion, and the act of giving birth, etc. The more children the women have early in life, the more they reduce their nutritional intake due to family resource constraints and the burden of raising them, and the fact that caring for children and the family is a very exhausting and physically demanding task, which prevents them from becoming obese and hypertensive, which in turn will reduce their risk of developing diabetes. When the number of children is increased, this means that urban mothers are under more pressure to raise their children, which in turn reduces their risk of diabetes.

For this reason, to better protect women's health after childbirth, it is necessary, on the one hand, to continuously improve the supporting policies to help women cope with the worries of childbirth, to reduce their stress in life and parenting, and to compensate for the loss of health caused by childbirth. On the other hand, publicity and education should be strengthened to provide more knowledge on scientific and rational diet and lifestyle habits to help them develop a correct concept of life and a scientific understanding of the act of childbirth and its consequences, so that they can be well-prepared to face the childbirth.

## Data Availability Statement

The raw data supporting the conclusions of this article will be made available by the authors, without undue reservation.

## Ethics Statement

The studies involving human participants were reviewed and approved by the Research Ethics Committee of Peking University (permission: IRB00001052−13074). The patients/participants provided their written informed consent to participate in this study.

## Author Contributions

Y-wG and SZ conceived this research. H-lY, J-hW, and S-qZ were responsible for the methodology. H-lY and SZ conducted software analyses. S-qZ, Y-dY, and Y-yW conducted necessary validations. LX conducted a formal analysis and managed the investigation. Z-yL, Y-dY, and J-yC gathered the resources, curated all data, wrote or prepared the original draft, and were responsible for project administration. SZ and H-lY reviewed and edited the manuscript, were responsible for visualization, supervised the project, acquired funding, and conducted software analyses. All authors contributed to the article, approved the submitted version, and reviewed the manuscript.

## Funding

This work was supported by the Later Funded Projects of National Social Science Foundation (grant number: 21FRKB003).

## Conflict of Interest

The authors declare that the research was conducted in the absence of any commercial or financial relationships that could be construed as a potential conflict of interest.

## Publisher's Note

All claims expressed in this article are solely those of the authors and do not necessarily represent those of their affiliated organizations, or those of the publisher, the editors and the reviewers. Any product that may be evaluated in this article, or claim that may be made by its manufacturer, is not guaranteed or endorsed by the publisher.
